# Emerging roles of Nrf2 signal in non-small cell lung cancer

**DOI:** 10.1186/s13045-016-0246-5

**Published:** 2016-02-27

**Authors:** Yijun Tian, Qian Liu, Xuelian He, Xun Yuan, Yuan Chen, Qian Chu, Kongming Wu

**Affiliations:** Department of Oncology, Tongji Hospital of Tongji Medical College, Huazhong University of Science and Technology, Building 303, 1095 Jiefang Avenue, Wuhan, 430030 People’s Republic of China; Clinical Research Center, Wuhan Medical and Healthcare Center for Women and Children, Wuhan, 430030 People’s Republic of China

**Keywords:** Nrf2, Keap1, Non-small cell lung cancer, MicroRNA, Ho-1, Nqo1

## Abstract

Non-small cell lung cancer (NSCLC) causes considerable mortality in the world. Owing to molecular biological progress, treatments in adenocarcinoma have evolved revolutionarily while those in squamous lung cancer remain unsatisfied. Recent studies revealed high-frequency alteration of Kelch-like ECH-associated protein 1/nuclear factor erythroid 2-like factor 2 (Keap1/Nrf2) pathway within squamous lung cancer, attracting researchers to focus on this particular pathway. In NSCLC patients, deregulated Nrf2 signal is recognized as a common feature at both DNA and protein level. Emerging associations between Nrf2 and other pathways have been elucidated. MicroRNA was also implicated in the regulation of Nrf2. Agents activating or antagonizing Nrf2 showed an effect in preclinical researches, reflecting different effects of Nrf2 during tumor initiation and progression. Prognostic evaluation demonstrated a negative impact of Nrf2 signal on NSCLC patients’ survival. Considering the importance of Nrf2 signal in NSCLC, further studies are required in the future.

## Background

Non-small cell lung cancer (NSCLC) remains to be the leading cause of tumor-related mortality [1, 2]. Among main pathological types of NSCLC, identification of epidermal growth factor receptor (EGFR) mutation [3, 4], echinoderm microtubule-associated protein-like anaplastic lymphoma kinase (EML4-ALK) fusion [5, 6], and other genetic alterations bring revolutionary improvements to the treatment of advanced lung adenocarcinoma. Other genetic/epigenetic alterations, including long non-coding RNAs HOTAIR [7] and GAS5 [8] and potential oncogenes Notch1 [9], alpha-enolase [10], and NLK [11] are also contributed to the progression of NSCLC. Biomarker-guided strategy has been demonstrated to improve chemotherapy response for NSCLC patients [12]. However, conventional chemotherapy and radiotherapy continue to be the standard regime for squamous lung cancer patients who lose their chances to surgery [13].

To better understand the genetic feature of squamous cell lung cancer, The Cancer Genome Atlas (TCGA) network attempted to unveil the genomic alterations in this common pathological type through comprehensive approaches [14]. Kelch-like ECH-associated protein 1 (Keap1)/nuclear factor erythroid 2-like factor 2 (Nrf2)/Cullin3 pathway alterations occur in a third of squamous cell lung cancer according to TCGA discoveries. Another study conducted by Kim et al. indicated that the proportion in East Asian population was as high as 39.4 % [15]. Keap1 negatively regulates intracellular Nrf2 protein abundance and represses the activation of Nrf2 signal [16]. Gene knockout mice model and clinical studies proved that Nrf2 signal is crucial in the initiation and progression of lung cancer. Nrf2 signal exerts a favorable chemopreventive influence on mice teratologenic tests by promoting carcinogen elimination, suggesting its anti-initiation effects [17]. Clinical observations also suggested a correlation between enhanced Nrf2 signal activities and worse treatment outcomes [18]. Expressions of various cytoprotective genes are upregulated when Nrf2 signal activates to increase pro-survival potential under endogenous or exogenous stress stimulation [19]. These genes are involved in multiple biological processes including glutathione synthesis, purine denovo synthesis, glycometabolism, drug-pump system, and serine synthesis [20–23]. Furthermore, there are crosstalks between Nrf2 and other oncogenic signal pathways such as phosphatidylinositol 3-kinase (PI3K) [24], Kirsten retrovirus-associated DNA sequence (K-ras) [25], and Notch [26]. This minireview will mainly focus on emerging relevance between Nrf2 signal and NSCLC to give a glimpse of what have been achieved in this realm.

## Nrf2 and Keap1 expression in NSCLC

Tobacco exposure is considered to be the principal cause of non-small cell lung cancer [27]. As a major carcinogen for squamous cell lung cancer, cigarette exposure can activate the oxidant stress response [28]. Sekine et al. analyzed gene expression of H292 (human lung mucoepidermoid cancer cell) and found that after exposure to total particular matter (TPM) of tobacco leaf, Nrf2-mediated oxidative stress response was significantly activated [29]. Hu et al. examined Nrf2 sequences of 103 patients with NSCLC and discovered that the Nrf2 mutation rate in ever-smokers was significantly higher than that in never-smokers [30]. In accordance with Hu, Sasaki et al. sequenced Nrf2 in 262 surgically resected lung tumors and confirmed that Nrf2 mutation were more common in squamous lung cancer and smokers [31]. Genomic analysis also showed approximately 30 % of squamous lung cancer harbor alterations within the Keap1/Nrf2 pathway [14, 15].

On the other hand, Singh et al. demonstrated that deletion of Keap1 locus (19p13.2) recurrently occurred in NSCLC, which might increase the nuclear accumulation of Nrf2 and reduce tumor’s sensitivity to chemotherapy [32]. In addition, Muscarella et al. discovered that 22 in 47 NSCLC exhibited a hypermethylation of CpG in Keap1 promoter [33].

At protein level, several studies have shown that Nrf2 was frequently deregulated in NSCLC tumor tissues [18, 34]. Solis et al. demonstrated that nuclear Nrf2 abundance was higher in squamous cell lung cancer than in adenocarcinoma [35]. Keap1 absent or low in abundance were more common in adenocarcinoma. And it was indicated that nuclear Nrf2 abundance associated with worse progress-free survival in squamous lung cancer patients treated by platinum-based adjuvant regimen.

Increasing numbers of microarray assays have been conducted to profile NSCLC genomic features, which provide an opportunity to link novel target genes with clinicopathological characteristics. Cescon et al. reanalyzed squamous lung cancer’s expression profile of TCGA and two other datasets to identify a gene list associated with Nrf2 activation, and eventually separated squamous lung cancer into activated and wild-type groups [36]. This molecular signature classification was reproducible and could help predict survival within certain studies to some extent.

## Interactions between Keap1 and Nrf2

**Fig. 1:**
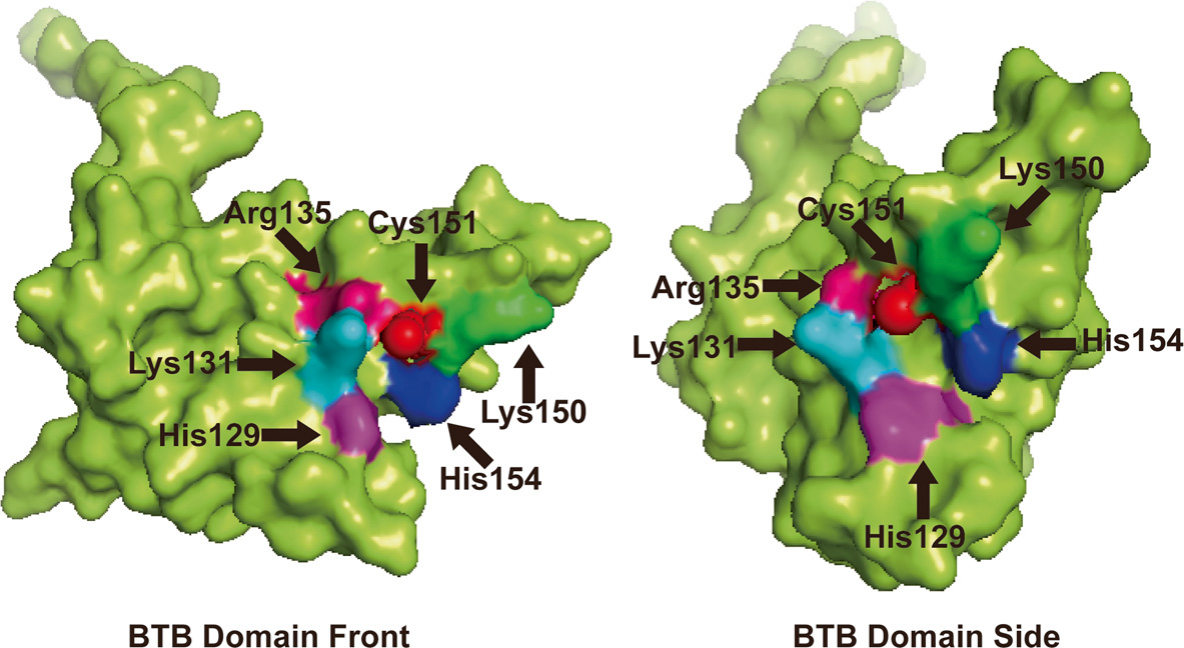
Corresponding amino residuals within Keap1 BTB domain on tertiary structure. Amino residues marked in different colors with *arrows* showed its serial number on peptide chain. Simulation of tertiary structure was constructed using PDB file of 4CXI produced by Cleasby et al. [110]. PyMOL Molecular Graphics System was used to present this domain

Keap1/Nrf2 pathway modulates redox homeostasis in mammal cells [37]. Nrf2 contains a basic-leucine zipper structure and belongs to the Cap’n’Collar transcription factors [38]. By linking its ETGE and DLG motifs with dimerized Kelch domain, a model called “hinge and latch” is fixed to the actin cytoskeleton [39]. As a negative regulator of Nrf2, Keap1 assembles Cullin3 to form Cullin-E3 ligase complex which degrades Nrf2 protein via ubiquitin-proteasome route [34]. When electrophiles and xenobiotics appear intracellularly, bounds between Nrf2 and Keap1 are counteracted [40]. Nrf2 protein then evades degradation and translocates from cytoplasm to nucleus under the direction of a bipartite nuclear localization signal (NLS) [41, 42], thereby dimerizing with c-Jun [43] and small Maf [44] before binding to the antioxidant response element (ARE) [45]. It has been demonstrated that amino residuals on Keap1 protein directly react with electrophiles and xenobiotics to perceive intracellular stress condition [46–49]. Table 1 summarized Keap1 amino residuals involved in the activation of Nrf2. Figure 1 illustrated tertiary structure of Broad complex, Tramtrack, and Bric à brac (BTB) domain of Keap1.

**Table 1:** Summary of Keap1 amino residuals involved in the activation of Nrf2 signal

Author	Interests amino residues	Nrf2 signal activator
2003 Zhang et al. [46]	Cys151, Cys273, Cys288	Sulforaphane, t-BHQ
2002 Dinkova-Kostova et al. [47]	Cys257, Cys273, Cys288, Cys297, Cys613	Dexamethasone, sulforaphane
2010 McMahon et al. [49]	Cys288, His225, Cys226, Cys613, His129, Lys131, Arg135, Lys150, His154, Cys151	NO, Zn2+, alkenals
2014 Wang et al. [48]	Cys151	Oxaliplatin

Nrf2 downstream genes generally contain a conserved sequence in the promoter region, which binds with Neh4 and Neh5 domain of Nrf2 [50]. ARE exists in a variety of intracellular antioxidant genes such as glutamate-cysteine ligase modifier subunit (Gclm), NAD(P)H quinone oxidoreductase 1 (Nqo1), glutathione S-transferase (Gst), heme oxygenase-1 (Ho-1) [51]. These genes encode phase II metabolic enzymes which mainly participate in the defense of drugs and reactive oxygen species (ROS) [52]. Gclm is a rate-limiting enzyme involved in the synthesis of glutathione [38]. Gst is best known for its ability to catalyze the conjugation of GSH with xenobiotics substrate, which can help in detoxification. Nqo1 catalyzes the process of NAD(P)H dehydrogenation to NAD(P)+. After the dehydrogenation, a quinone turns into a hydroquinone which could be easily eliminated in water-soluble form [53]. Different from the above three genes, Ho-1 plays an important role in attenuating inflammatory response and preventing cell apoptosis. Ho-1 could bind to gene promoter as well as directly interact with inflammation factor Stat3 besides its heme degradation function [54]. Dey et al. demonstrated that Ho-1 prevented anoikis (a special form of apoptosis) and promote metastasis of colorectal fibrosarcoma cells [55]. However, Ho-1 exhibited an unusual antitumor effect in mucoepidermoid lung carcinoma by down-regulation of matrix metalloproteinase [56, 57]. In addition, Multidrug resistance-associated protein 1 (MRP1) contains two potential AREs which may interact with Nrf2 when its activator tertiary butylhydroquinone (t-BHQ) is administrated to small cell lung cancer cell line H69 [58].

Recently, the involvement of Nrf2 has also been recognized in mitochondrial physiology [59]. Through producing more substrates (NADH and FADH2) for respiration and augmenting aliphatic acid oxidation, Nrf2 influences mitochondrial activity [60]. Keap1/Nrf2 signal regulated both mitochondrial and cytoplasmic ROS production through NADPH oxidizing in cortical neurons and glial cells [61]. Besides, Nrf2 affected other physiological characteristics of mitochondrion including membrane potential [62], membrane integrity [63], and biogenesis [64].

## Emerging gene crosstalks with Nrf2 signal

Classical oncogenic pathways such as PI3K and K-ras have been reported to have an impact on Nrf2 function, as well as some other well-known transcription factors such as Bach1, estrogen receptor(ER)-α, NF-kappa B, and HIF-1α.

### Nrf2 and PI3K

PI3K signal pathway is a classical oncogenic gene as it enhances tumor cell growth, viability, and metabolism [65]. PI3K inhibitor NVP-BKM120 reduced expression of Nrf2 in squamous lung cancer cells [24]. However, the mechanism involved has not been elucidated. Activated PI3K signal increased Nrf2 accumulation in nuclear [21], thereby enhancing multiple biological processes including de novo purine nucleotides synthesis, glutamine metabolism, and pentose phosphate pathway. Among these processes, enzymes involved in the pentose phosphate pathway provided substrates for purine synthesis and glutamine metabolism to promote cell proliferation and cytoprotection.

### Nrf2 and K-ras

K-ras gene mutations repeatedly occur at a proportion of 20~30 % in NSCLC [66]. Mutated K-ras proteins cause aberrant activation of downstream signal and confer to cancer cells’ resistance and survival. Lung adenocarcinoma patients harboring K-ras mutation tended to be chemoresistant and had dismal prognosis [67, 68]. Tao [25] and DeNicola et al. [69] identified that constitutive expression of K-ras mutation G12D enhanced Nrf2 mRNA levels. Promoter analysis showed that a TPA response element (TRE) located in exon1 of Nrf2 was activated by K-ras. Remarkably, Satoh et al. modeled the process of lung carcinogenesis with urethane and found that Nrf2^−/−^ mice were rarely associated with K-ras mutation [17]. They also established Nrf2 prevented tumor initiation but promoted progression in different phases during carcinogenesis.

### Nrf2 and Bach1

Bach1, a nuclear transcription factor, was reported to co-localize with Nrf2 in nucleus in HepG2 cells and attenuate the binding between Nrf2 and ARE [70]. This negative regulation of Bach1 resulted in the balance of redox within cells. In earlier research of Sun et al., evidences revealed that the repression was mediated by Ho-1 and its substrates heme [71]. Reichard et al. found that during arsenite-mediated oxidative stress, Bach1 inactivation allowed Nrf2 binding to Ho-1 promoter and elevating Ho-1 mRNA [72].

### Nrf2 and ER-α

Estrogen receptor (ER) is tightly related to the development and biological behavior of multiple cancers. Researches suggested that ER-α repressed the activity of Nrf2 and the transcription of phase II metabolic enzymes [73, 74]. Further exploration revealed that this repression resulted from the interaction between ER-α and Nrf2 and required the coordination of ER ligand 17-estradiol [73].

### Nrf2 and Sirt1

Acetylation of amino residuals typically stabilized Nrf2 proteins and prevented it from degradation [75]. Sirt1 is an enzyme primarily engaged in catalyzing protein deacetylation in nucleus [76]. Kawai et al. noticed that CREB-binding protein (CBP) mediated acetylation of Nrf2 and gave rise to its target gene mRNA, while Sirt1 deacetylated Nrf2 and vice versa [77]. By constructing mutations of pK588Q and pK591Q, they unveiled an indispensible role of lysine residuals on Nrf2 in the process of Sirt1 regulation.

### Nrf2 and NF-kappa B

Inflammatory response activation always occurs with elevation of ROS [78]. As a classical pro-inflammatory factor, NF-kappa B has been implicated in the regulation of Nrf2. Liu et al. found that NF-kappa B subunit p65 specifically deprived CBP from Nrf2, leading to inhibition of Nrf2 and its downstream genes [79]. Oppositely, Rushworth et al. recently reported that NF-kappa B subunits p50 and p65 promoted transcription of Nrf2 by binding to a kappa B site in acute myeloid leukemia, and conferred to resistance to cytotoxic treatment [80]. These findings suggested distinct patterns of crosstalk between NF-kappa B and Nrf2 in different cell contexts.

### Nrf2 and HIF-1α

HIF-1α is a key transcription factor mainly monitoring oxygen homeostasis. Under hypoxic condition, HIF-1α escapes from degradation mediated by prolyl hydroxylase domain proteins and augments downstream gene expression [81]. In human endothelial cells, Loboda et al. discovered that induction of HIF-1α attenuated Nrf2-dependent expression of IL-8 and Ho-1 [82]. Thereafter, investigator in the realm of colon cancer has identified Nrf2 as an important factor in activating HIF-1α. Kim et al. found that stably inhibiting Nrf2 signal in colon cancer cell led to attenuated HIF-1α activation, subsequently causing a reduction of blood vessel formation and vascular endothelial growth factor expression [83].

### Nrf2 and Notch1

Notch family consists of a series of intracellular signal mediators with highly conserved domain [84, 85]. It was reported that Notch1 and Notch3 expressions were closely associated with NSCLC patients’ progression and prognosis [86]. Wakabayashi et al. found Notch signal activation upregulated Nrf2 and cytoprotective genes in mouse liver [87]. They also demonstrated that Notch intracellular domain (NICD) assembled to the Rbpjκ site of Nrf2 promoter, leading to the activation of Nrf2 signal. Inversely, Nrf2 activation induced by ROS enhanced the Notch pathway, thus promoting airway basal stem cells’ self-renewal [88]. Paul et al. identified a putative ARE within Notch1 promoter [88]. More recently, Zhao et al. discovered that ionizing radiation exposure induced Nrf2 activation and knockdown of Nrf2 attenuated Notch1 expression following ionizing radiation [89]. The evidences above indicated a mutual promotion model for the crosstalk of Nrf2 and Notch1.

## MicroRNAs associated with Nrf2

MicroRNA-related mechanisms play a critical role in the regulation of Nrf2. Several studies have identified microRNAs which directly decreased Nrf2 mRNA in breast and esophageal cancer. miR-28 targeted 3′-untranslated region (UTR) of Nrf2 to exhibit a significant silencing effect in breast cancer [90]. In addition, by screening reporter-coupled microRNA library, Yamamoto et al. discovered that miR-507, miR-634, miR-450a, and miR-129-a directly targeted Nrf2 to mediate mRNA degradation in esophageal cancer [91]. Besides, miR-200a was reported to associate with and trim Keap1 mRNA and thus increased the levels of Nrf2 protein and downstream transcripts [92].

Nrf2 also modulates microRNAs to mediate pro-survival processes. In lung cancer, Singh et al. further examined Nrf2’s effects on the pentose phosphate pathway and tricarboxylic acid cycle, discovering that activation of Nrf2 reduced miR-1 and miR-206 expression and resulted in elevation of metabolic gene expression in the pathway [93]. Chemotherapy induces apoptosis in not only cancer cells but also normal tissue. Joo et al. reported that oltipraz, a synthetic Nrf2 activator, increased miR-125b in the kidney of mice [94]. miR-125b subsequently inhibited the activity of aryl hydrocarbon receptor repressor, leading to augmentation of Mdm2 and reduction of p53, thus to protect the kidney against acute injury caused by cisplatin. Table 2 gives a summary of microRNAs associated with Nrf2 signal.

**Table 2:** Lists of micRNAs associated with Nrf2 signal

	MicroRNA ID	Target region/biological process involved	Organ types
Increased by Nrf2	miR-125b [94]	Inhibit AhR repressor	Kidney, liver
Decreased by Nrf2	miR-1, miR-206 [93]	Pentose phosphate pathway, tricarboxylic acid cycle, glucose metabolism	Lung
Increase Nrf2	miR-200a [92]	Keap1 mRNA’s 3′-UTR	Breast, liver
Decrease Nrf2	miR-28 [90]	Nrf2 mRNA’s 3′-UTR	Breast
	miR-507, miR-634, miR-450a, miR-129-5a [91]	Nrf2 mRNA’s 3′-UTR	Esophageal

*UTR* untranslated regions

## Typical activators and antagonists of Nrf2 signal

Activators of Nrf2 signal have long been studied for their effects in inducing detoxication and cytoprotective genes, generating a chemopreventive effect towards carcinogenesis. Among thousands of newly synthetic or extracted compounds, typical activators of Nrf2 commonly derive from plants such as broccoli [95] and turmeric [96].

*Sulforaphane*, which is extracted from broccoli, is one of the most potent activators of Nrf2 signal. Hong et al. demonstrated that sulforaphane modified Kelch domain of Keap1 protein [97]. Thiols from Kelch domain react with isothiocyanate on sulforaphane to form a thionoacyl adduct, releasing Nrf2 protein and inducing phase II metabolic enzymes. Kalpana et al. tested the ability of sulforaphane in inhibiting benzo(a)pyrene (B(a)P)-initiated lung carcinogenesis in mouse and confirmed its impact on Nrf2 signal pathway [98]. Intriguingly, sulforaphane could induce apoptosis through ROS-mediated mitochondrial pathway [99].

*Curcumin*, which is extracted from an Indian spice named turmeric, is also a classical Nrf2 signal activator. A series of studies emphasized its radiation-protective or chemoprevention role in normal tissues and indicated the protective effects are mediated by activating Nrf2 signal [96, 100]. Intriguingly, curcumin yet can act as a radiotherapy/chemotherapy sensitizer in colorectal cancer [101, 102], prostate cancer [103], and ovarian cancer [104]. It is remarkable that curcumin also has an inhibitory effect on other oncogenic signal pathways such as NF-kappa B [104], Notch1 [105], and mitochondrial pathway [106], therefore providing more rationale for its clinical practice in the future.

*Oltipraz*, known as a dithiolthione substitute capable of inducing phase II enzymes, exhibited a chemoprevention effect [107]. Lida et al. demonstrated that Nrf2 was responsible for oltipraz’s chemoprevention effect against bladder carcinogenesis [108]. Sharma et al. proved that inhalation of oltipraz as spray inhibited B(a)P-initiated lung adenocarcinoma in mouse [109].

*CDDO-Im* is another powerful activator of Nrf2 signal. It is a synthetic oleanolic triterpenoids that can covalently conjugate with electron-withdrawing groups. Cleasby et al. identified CDDO-Im covalently formed complex with Keap1 on BTB domain [110]. This complex inhibited the binding of Keap1 BTB domain and Cullin3 to activate Nrf2 signal. By applying microarray to Keap1-knockout and CDDO-Im disposed mice, Yates et al. demonstrated that both methods exerted a comprehensive activation of Nrf2-regulated gene [111]. In vivo evidence suggested an oral dose of 1~100 μM/kg CDDO-Im protected hepatic cells against aflatoxin-induced tumorigenesis [112].

As to antagonists of Nrf2 signal, limited compounds were identified to exhibit obvious inhibitory effect. Brusatol is a quassinoid firstly reported to have an antitumor effect for leukemia [113]. Ren et al. found that brusatol enhanced ubiquitination and degradation of Nrf2 and reduced its protein level [114]. Pretreatment with brusatol increased cancer cells’ sensitivity to chemotherapy. In mouse xenograft model, brusatol combined with cisplatin significantly reduced expressions of Nrf2, Nqo1, and Ki-67 indexes. Research also suggested that the inhibitory effect caused by brusatol was a transient process which happened within 12 h after its administration [115]. Posttranscriptional regulation was recognized as the main inhibition mechanism.

## Prognostic value of Nrf2 signal in NSCLC

As introduced above, Nrf2 and its downstream transcripts protect cells against exogenous stimuli and oxidant stress, thus increasing lung cancer cells’ resistant to antineoplastic treatment. Inoue et al. examined the expression of Nrf2 by immunohistochemical in 109 NSCLC specimens and discovered that higher nuclear accumulation of Nrf2 correlated with worse lung cancer-specific survival [116]. Solis et al. further explored nuclear Nrf2 and cytoplasm Keap1 immunohistochemical expression in 304 NSCLC patients and reported that nuclear Nrf2 expression associated with worse progress-free survival in squamous cell cancer patients who underwent adjuvant treatment [35]. Yang et al. analyzed Nrf2 abundance of 60 NSCLC patients and compared platinum-based treatments response between patients with <75 % positive stain and that with 75–100 % positive stain [18]. It was discovered that the former group achieved a higher response rate than the latter group, suggesting that Nrf2 expression might be a useful index to predict the efficacy of platinum-based treatments.

As the main negative regulator of Nrf2, Keap1 activity also correlated with NSCLC survival. Muscarella et al. discovered that NSCLC patients harboring both Keap1 somatic mutation and methylation had worse progress-free survival compared with other patients [33]. Similarly, Takahashi et al. found that Keap1 mutations conferred to the increase of Nrf2 abundance in NSCLC patients and worse progress-free and overall survival [117].

With regard to Ho-1, one of Nrf2 downstream transcripts, correlation between its expression and survival has not yet been elucidated. Degese et al. pointed out that Ho-1 expression correlated with advanced stage and lymphatic metastasis, but no associations with patients’ overall survival were found [118]. In study of Tsai et al., Ho-1 expression in 70 NSCLC tumor tissues were assessed with matched normal tissues [119]. The results indicated that patients with a Ho-1 mRNA rise (defined as ratio between tumor and normal bigger than 1) exhibited worse overall survival and higher metastasis rate.

Another important transcript of Nrf2 signal, Nqo1, encoding a flavoprotein previously named DT-diaphorase and mainly acting as a catalyzer of oxidation of NA(D)PH, predicted NSCLC survival at different levels. Early before, Pamela et al. related DT-diaphorase expression and activity in NSCLC tumors to smoking status [120]. Then, Kolesar et al. validated that expressions of Nqo1 in lung tumors were higher than the matched normal lung tissues [121]. Moreover, they also evaluated Nqo1 single nucleotide polymorphism (SNP) by restriction fragment length polymorphism (RFLP), and found that homozygous SNP genotype was associated with worse overall survival [122]. Recently, Li et al. also demonstrated that patients with positive expression of Nqo1 stain in tumors have shorter overall survival [123].

## Conclusions

**Fig. 2:**
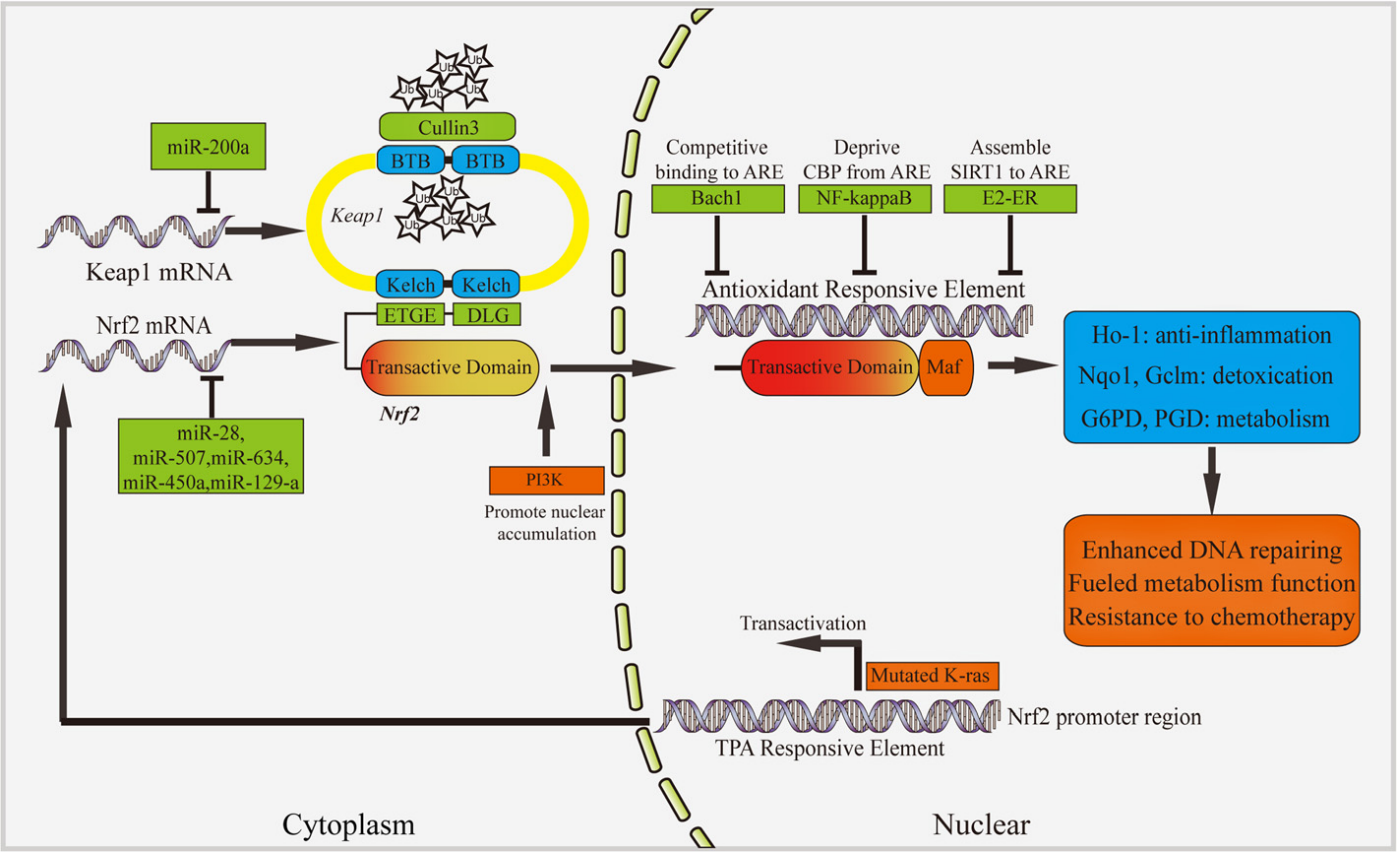
Schematic illustration of pathways associated with Nrf2 signal. Keap1 assembles Cullin 3 and binds to the ETGE and DLG sites of Nrf2 through Kelch domain, leading to the degradation of Nrf2. In the absence of Keap1, Nrf2 translocates from cytoplasm to nuclear to bind with ARE in the promoter region of target gene, leading to the transcriptional activation of genes related to inflammation, detoxication, and metabolic regulation. However, Nrf2 activity could be modified by acetylation and deacetylation through NF-kappa B or ER pathway. Nrf2 activity could also be inhibited by Bach1 through competitively binding with ARE. Mutant K-ras promotes Nrf2 transcriptional through TPA responsive element. Several microRNAs have been shown to inhibit Nrf2 or Keap1. *BTB* Broad complex, Tramtrack, and Bric à brac, *ARE* antioxidant responsive element, *ER* estrogen receptor, *Ub* ubiquitin, *CBP* CREB-binding protein

Although Nrf2 has been newly identified as oncogenic signal pathway, it has not been proved to be a driver gene in NSCLC. Nrf2 signal is inextricably linked to classical oncogenic pathways (Fig. 2). MicroRNA played an important role in regulation of Nrf2 signal. Both activators and antagonists towards Nrf2 have been applied in preclinical researches, reflecting its two-side effect during lung tumor initiation and progression. Yet, the effect requires more evidences before putting into clinical practice. Nrf2 signal is characterized as a potential biomarker in NSCLC progress and prognosis.

## Abbreviations

ARE: antioxidant response element
B(a)P: benzo(a)pyrene
BTB: Broad complex, Tramtrack, and Bric à brac
CBP: CREB-binding protein
EGFR: epidermal growth factor receptor
EML4-ALK: echinoderm microtubule-associated protein-like anaplastic lymphoma kinase
ER: estrogen receptor
Gclm: glutamate-cysteine ligase modifier subunit
Gst: glutathione S-transferase
Ho-1: heme oxygenase-1
Keap1: Kelch-like ECH-associated protein 1
K-ras: Kirsten retrovirus-associated DNA sequences
MRP1: multidrug resistance-associated protein 1
NICD: Notch intracellular domain
Nqo1: NAD(P)H quinone oxidoreductase 1
Nrf2: nuclear factor erythroid 2-like factor 2
NSCLC: non-small cell lung cancer
PI3K: phosphatidylinositol 3-kinase
RFLP: restriction fragment length polymorphism
ROS: reactive oxygen species
SNP: single nucleotide polymorphism
t-BHQ: tertiary butylhydroquinone
TCGA: The Cancer Genome Atlas
TPM: total particular matter
TRE: TPA response element
UTR: untranslated region
